# An unusual intraoral lipoma: case report and review of the literature

**DOI:** 10.11604/pamj.2022.41.336.34808

**Published:** 2022-04-26

**Authors:** Youssra Azzouz, Soukaina Abidi, Fatima Zahra Zidane, Saliha Chbicheb

**Affiliations:** 1 Oral Surgery International Faculty of Dentistry, International University of Rabat, Rabat, Morocco,; 2 Pediatric Dentistry International Faculty of Dentistry, International University of Rabat, Rabat, Morocco,; 3 Faculty of Dentistry, Mohammed V University, Rabat, Morocco

**Keywords:** Oral lipoma, buccal mucosa, treatment, case report

## Abstract

Lipoma is a common tumor of soft tissue with rare occurrence in oral cavity accounting for only 1-4% of benign oral tumours. It may be noticed only during routine dental examinations. Most of them rarely cause pain, resulting in delay to seek treatment. Lipoma of the oral cavity may occur in any region. The buccal mucosa, tongue, and floor of the mouth are among the common locations. A case of large intraoral lipoma occurring in mental region in a 60-year-old female patient is reported. It was treated surgically under local anesthesia, and 6 month follow up showed excellent healing without any recurrence.

## Introduction

Odontogenic lipomas are very common benign tumor of mesenchymal origin of human body composed of mature adipocytes and usually surrounded by a thin fibrous connective tissue capsule [1,2]. They commonly occur in the head and neck region; however, 1-4% affect the oral cavity [3]. Lipomas particularly occur in areas of fat accumulation, in especially, cheeks, followed by the tongue, the floor of the mouth, buccal sulcus and vestibule, lip, palate, and gingiva [3]. Oral lipomas have shown no gender predilection. Some studies, however, have reported a male predilection. Lipoma is uncommon in children and affected patients mostly are 40 years of age or older [4,5].

The aetiology and pathogenesis remain unclear although mechanical, endocrinal endocrine disorders, obesity, hypercholesterolemia, radiation, and influences of chromosomal abnormalities and inflammatory influences have been reported [6,7]. The aim of this article is to present a case of an adult female patient with intraoral lipoma that was treated by surgical excision with no recurrence or complications.

## Patient and observation

**Patient information:** a 60-year-old female patient was referred to the Department of Oral Surgery with the complaint of swelling in relation to lower left mental region for the past year. The swelling gradually increased in size to attain its present size. The patient also complained of her disability to wear her complete dentures and feeling of heaviness in the area of the swelling. Her medical history was unremarkable.

**Figure 1:**
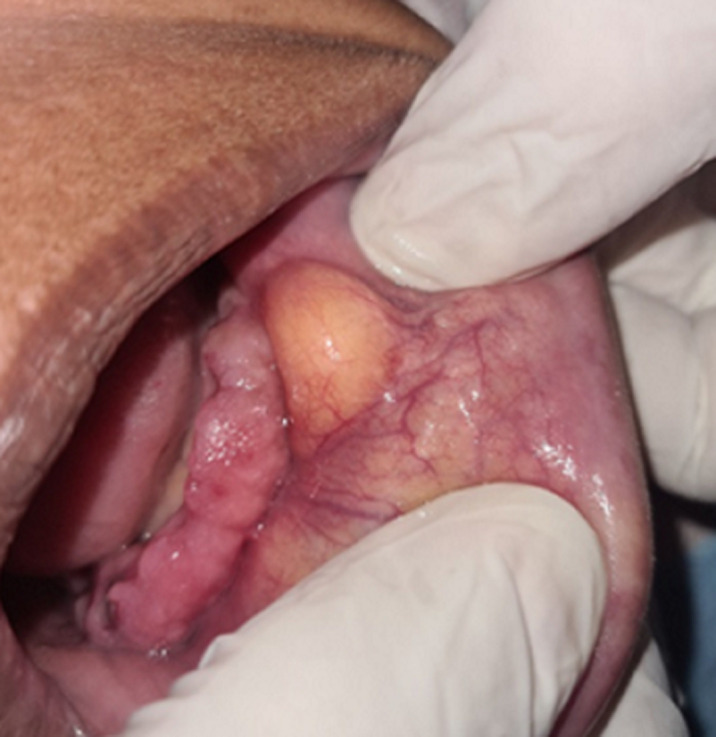
intraoral examination with tumefaction visible in the buccal mucosa and no signs of trauma

**Clinical findings:** extraoral examination revealed a diffuse swelling in the left lower mental region. On palpation, the swelling was mobile, firm, and nontender. Intraoral examination revealed a yellowish, oval swelling in the buccal left sulcus in relation to the 33,34,35 regions (Figure 1). The oral mucosa over the mass appeared normal without ulceration or inflammation. Based on history and clinical findings, a provisional diagnosis of lipoma was made.

**Figure 2:**
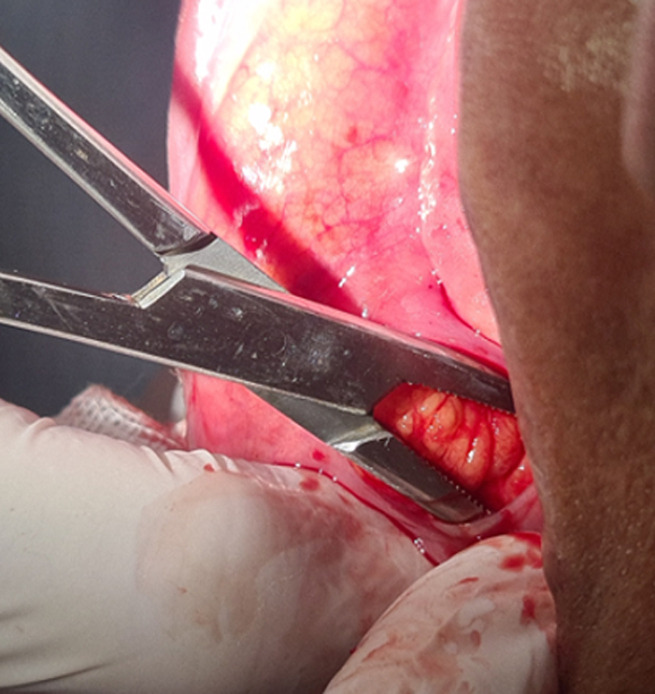
peripherical dissection with hemostatic forceps

**Figure 3:**
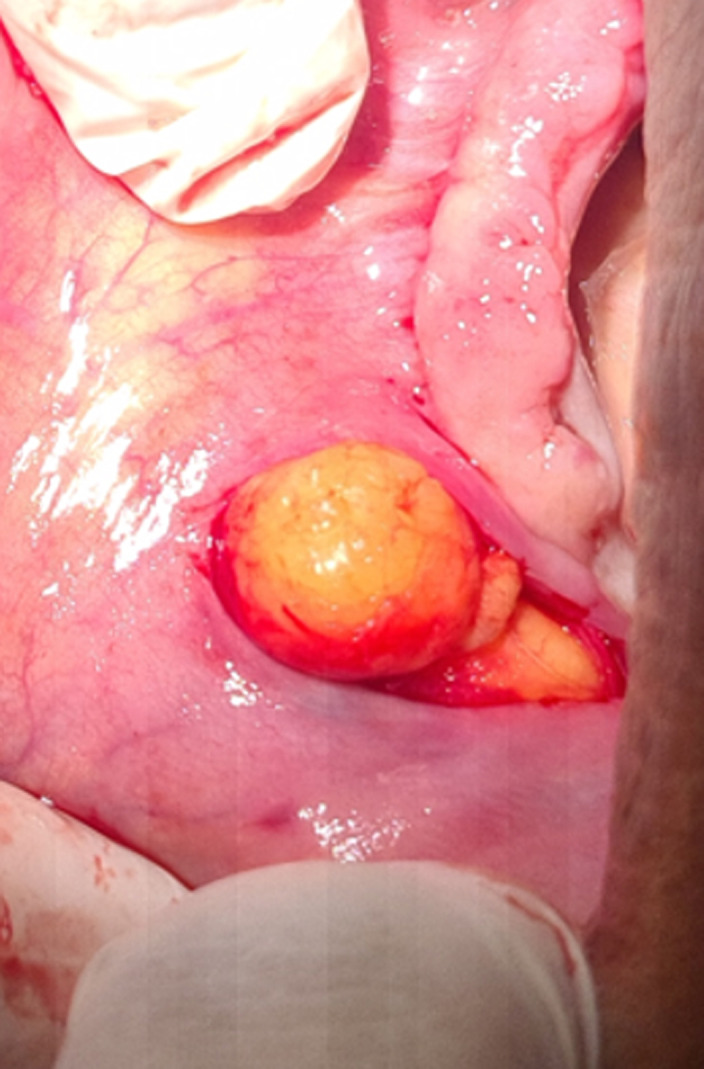
exposed lobulated, yellowish and poorly encapsulated mass

**Figure 4:**
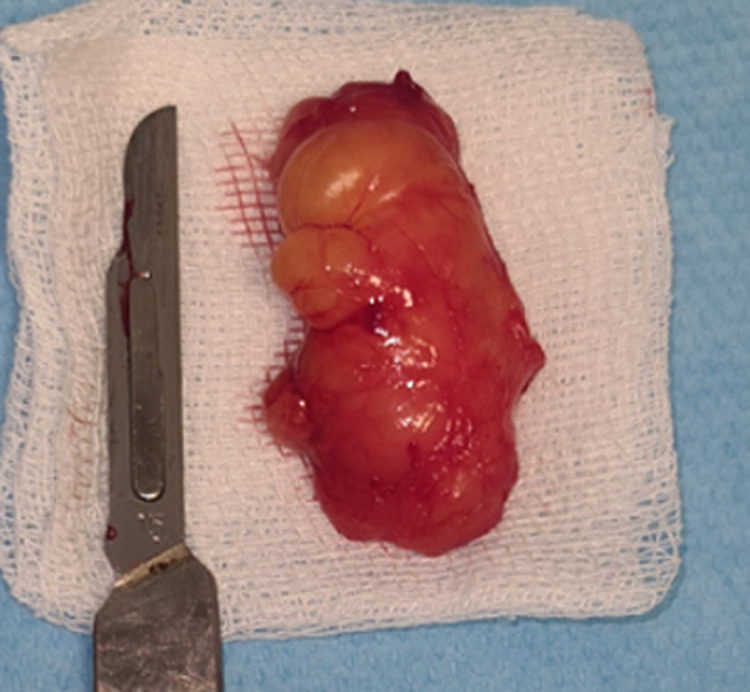
macroscopic image of the surgically excised tissue

**Therapeutic interventions:** excisional biopsy was performed under local anesthesia. Blunt dissection was performed (Figure 2); exposing an irregular, poorly encapsulated, and lobulated yellow mass (Figure 3). Excised specimen was 3.8cm in size (Figure 4). The mass was transferred to 10% buffered formalin and was sent for histopathological examination.

**Figure 5:**
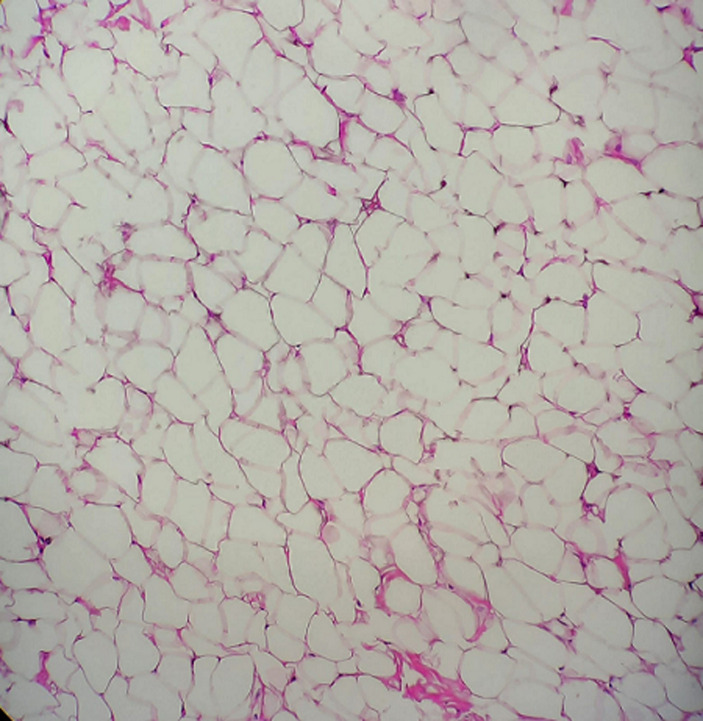
photomicrograph showing abundant mature adipocytes separated by fibrous connective tissue septa (x10)

**Figure 6:**
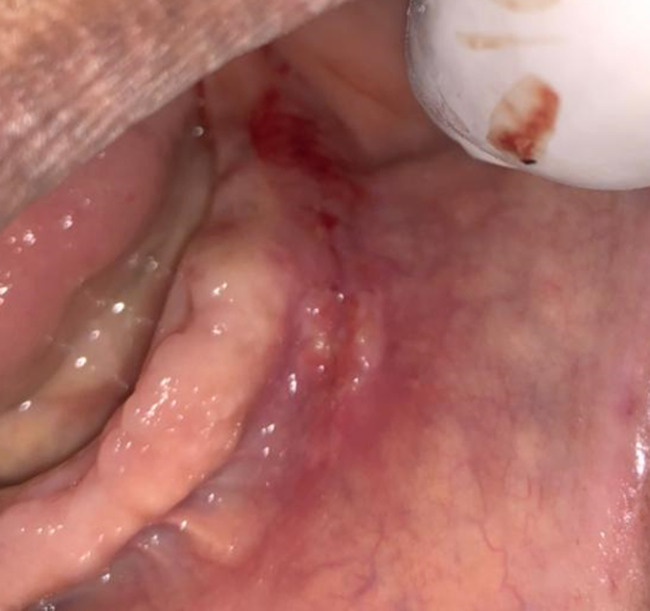
the 14^th^ day post-operation examination showed closure of the incision margin

**Follow-up and outcome of interventions:** the microscopic sections of the specimen stained with H&E showed a lobular arrangement composed of abundant mature adipocytes. The lobules are separated by fibrous connective tissue septa. The adipocytes were large round to ovoid cells with clear cytoplasm and eccentric nuclei compressed against the cytoplasmic membrane (Figure 5). At the borders, the lesion was surrounded by a thin fibrous capsule. According to histopathologic features, a definitive diagnosis of lipoma was made. A review after 7 days and 14 days showed uneventful healing and the sutures were therefore removed (Figure 6). Six months follow up showed excellent healing without any recurrence.

## Discussion

Lipomas are the most common soft tissue neoplasm but they are rare tumors of oral cavity, an uncommon sight for the occurrence of lipoma [1,3]. Lipoma can affect both soft and hard tissues. There are few cases that reported an intraosseous lipoma in the jaw.

Clinically, lipomas present as slow growing, sessile, asymptomatic, round to avoid submucosal nodules, with yellowish color [2,3,8]. The size may vary from 0.2 to 1.5 cm in diameter, its size rarely is greater than 2.5 cm [9]. Symptoms may include a discomfort and doughy feel [1,3]. Large tongue lipoma causes various functional problems like dysphagia, mastication and speech difficulties [10].

Lipomas may present as solitary or multiple lesions. [5] Multiple lipomas can be associated with syndromes such as Gardner´s syndrome, neurofibromatosis, Decrum´s disease, multiple familial lipomatosis, encephalocraniocutaneous lipomatosis, and Pai syndrome [3].

Lipomas have a microscopic appearance that is comparable to normal fat tissue. They are made up of mature fat cells, but they differ slightly in size and form from regular adipocytes, and they are slightly larger (up to 200 mm in diameter) and have a higher metabolism [6-11]. Sometimes the capsule may be missing or broken [6]. Since there is a histological similarity between normal adipocytes and lipoma cells, accurate clinical and surgical information is very important in making a definitive diagnosis [12]. The differential diagnosis of intraoral lipoma includes oral dermoid and epidermoid cysts, oral lymphoepithelial cyst, benign salivary gland tumour, mucocele, benign mesenchymal neoplasm, ranula, ectopic thyroid tissue, and lymphoma. Lesions appearing as swelling on the dorsum of the tongue usually mimic hemangioma, lymphangioma, rhabdomyoma, neuroma, or neurofibroma [3].

Lipoma is usually treated with complete surgical resection. After adequate excision, there is no recurrence but infiltrative lipoma tends to recur after inadequate excision due to the fact that they are not encapsulated like simple lipomas [3-13].

Medical management of lipomas includes injectable steroids to treat soft tissue lipoma, which causes adipose tissue atrophy and shrinks the tumor size. It´s indicated on lipomas that are less than 2,54 cm in diameter. A monthly injection of a 1: 1 mixture of lidocaine and triamcinolone acetonide into the central region of tumour [3-13].

## Conclusion

Intraoral lipomas are a rare entity, which may be noticed only during routine dental examinations. The diagnosis of oral lipomas is usually clinical and surgical but histopathology remains the gold standard.
